# Book Reviews

**Published:** 1853-09

**Authors:** 


*Elements of Health and Principles of Female Hygiene.* By E. J. Tilt, M. D., etc. Philadelphia: Lindsay & Blakiston. 1853. 12mo. pp. 436.

The author of this work is favorably known to the medical public by a treatise on ovarian diseases. The attention which he has given to the affections peculiar to females, eminently qualifies him to advise respecting the means of preventing them; and we do not doubt that the general circulation of this work would be productive of much good. Practitioners will do well to provide themselves with copies, both for their own instruction, and that they may enforce their prophylactic precepts by so good an authority as Dr. T.

---

*Atlas of Pathological Histology.* By Dr. GOTTLIEB GLUGE, Prof. of Physiology and Path. Anat. in the University of Bruxelles, etc., etc. Translated from the German by JOSEPH LEIDY, M. D., Pathologist to St. Joseph's Hospital, etc., etc. With three hundred and twenty figures, plain and colored, on twelve copperplate engravings. Philadelphia: Blanchard & Lea. 1853. Quarto, pp. 100.

We are grateful to Messrs. Blanchard & Lea, and the translator, for the republication of this interesting, useful, and elegant quarto; and we sincerely hope that it will be appreciated by the medical profession of this country sufficiently to encourage the issuing from the American press of other works of a similar character, a fair proportion of which, we hope, may be of indigenous origin. Pathological histology, in other words the minute history of pathological formations, is necessarily involved in the study of the processes of disease. Its relation to the latter is the same as that of normal histology, or general anatomy, to the study of the normal functions, or physiology. Modern investigations have shed much light on this province of inquiry, both in its physiological and pathological aspects. Indeed, the separation between the two is arbitrary, for, as the author remarks, "disease is nothing more than a physiological process modified by accidental causes."

That the reader may have a better idea of the scope of the work than the title conveys, we give a list of the several subjects of which it treats. They are as follows:

1. Introduction. Tables of the magnitude and weight of the organs of man in the normal and abnormal conditions.

These would have been more readily available for reference had the translator taken the trouble to have placed the equivalents in English weights and measurements, by the side of those of the French, which the author employs. We would suggest this as an important improvement in another edition.

2. Development of the elements of tissues, viz., cells in the exuded plasma of vessels, etc.
3. The elements of the tissues combined in perfect or imperfect tissues, and arranged according to the processes of disease.

4. Formation of the blastema: nutrition, secretion and inflammation, congestion, hyperæmia, stasis, inflammation and its terminations, formation of pus, etc., etc.
5. Histological metamorphosis of the blood, within, and exterior to blood-vessels.
6. Pyæmia.
7. Gangrene.
8. Observations on histology; calcification, atheroma, colloid, enteritis, typhoid, scarlatina, glanders, etc., etc.
9. Explanation of plates.

The plates are finely executed, and the getting up of the work is highly creditable to the publishers. The philosophic and truly practical physician, whose practice is based on the present state of pathological science, will feel himself richly reimbursed for the money and time invested in its purchase and perusal.

For sale by DERBY, ORTON & MULLIGAN.

---

*The Practice of Surgery.* By JAMES MILLER, F. R. S. E., F. R. C. S. E., etc., etc. Third American, from the second Edinburgh edition. Edited, with additions, by F. W. SARGENT, M. D., one of the Surgeons to Wills Hospital. Illustrated by three hundred and nineteen engravings on wood. Philadelphia: Blanchard & Lea. 1853. 8vo. pp. 720.

We give the editor's preface to the present edition of this highly esteemed practical treatise:—

"In preparing the present edition of Prof. Miller's treatise *on the practice of surgery*, the editor has endeavored, so far as he was able, to supply any omissions which the author may have accidentally made, and has suggested such amendments as appeared advisable.

"A large number of wood-cuts have been introduced into the reprint, chiefly to illustrate objects described but not figured by the author. They are taken in most instances from the last edition of Mr. Fergusson's *Practical Surgery*, from Mr. Lawrence's *Treatise on the Eye*, and from Professor Gross's excellent monograph *On the Diseases and Injuries of the Bladder*.

"The favor with which Professor Miller's contributions have been received, renders it unnecessary to pass any encomiums upon them, farther than to state that the second edition of the *Practice of Surgery*, of which the present issue is a copy, is a great improvement upon the first; and that, in connection with the volume on the *Principles*, it forms one of the best expositions of the present state of surgery in the English language."

For sale by Derby, Orton & Mulligan.

---

*A Practical Treatise on the Diseases of Children.* By J. Forsyth Meigs, M. D., Lecturer on Medicine in the Philadelphia Medical Association, etc., etc. Second edition, revised and enlarged. Philadelphia: Lindsay & Blakiston. 1853. 8vo. pp. 711.

The first edition of this treatise appeared two or three years ago as one of a series of publications entitled the "Medical Practitioner's and Student's Library." In the present edition it is issued as a distinct publication. We have heretofore expressed our estimation of the work. The author's experience since the date of the first edition, has enabled him to render it more valuable. In typographical appearance this edition is superior to the former. The fact that the first edition has been for some time exhausted, is evidence that we were not at fault in regarding it as a highly creditable production, and a valuable treatise for the practical physician.

For sale by Derby, Orton & Mulligan.

---

*On Diseases of the Liver.* By George Budd, M. D., F. R. S., etc. etc. Second American, from the last and improved London edition. With colored plates and wood-cuts. Philadelphia: Blanchard & Lea. 1853. 8vo. pp. 468.

This well known work has received, in the present edition, from the hands of the author, such modifications and improvements as continued clinical observations, and the late remarkable physiological discoveries pertaining to the liver, have suggested. It is, thus, an exponent of the present state of our knowledge of the diseases of that organ.

For sale by Derby, Orton & Mulligan.

---

*Essays on Asylums for Persons of Unsound Mind.* By John M. Galt, M. D., Sup't of Eastern Lunatic Asylum of Virginia.

Dr. Galt, in these two essays, endorses the human treatment of the insane now so generally adopted. He urges the utility of reading, and recreation, on their shattered minds, and employs all those humanizing means, which always mitigate, and frequently remove insanity. S. B. H.

*Principles of Medicine: comprising General Pathology and Therapeutics, and a brief general view of Etiology, Nosology, Semeiology, Diagnosis, Prognosis, and Hygienics.* By CHARLES J. B. WILLIAMS, M. D., etc., etc., etc. Edited, with additions, by MEREDITH CLYMER, M. D., etc. etc. Fourth American edition. Revised. Philadelphia: Blanchard & Lea. 1853. 8vo. pp. 476.

The rapid sale of three American editions of this work sufficiently attests the estimation in which it is held on this side of the Atlantic. The present edition embraces additions rendered necessary by the developments incident to the progress of medical science. The American editor has added much important matter. It is quite needless to commend it to the continued favor of the profession.

For sale by DERBY, ORTON & MULLIGAN.

